# Patient experiences in retinal trials: a cross-sectional study

**DOI:** 10.1186/s12886-015-0071-6

**Published:** 2015-07-23

**Authors:** Cheryl Pui-Yan Au, Nicole Fardell, Maria Williams, Samantha Fraser-Bell, Anna Campain, Mark Gillies

**Affiliations:** Department of Ophthalmology, Westmead Hospital, Sydney, Australia; Sydney Medical School, University of Sydney, Sydney, Australia; Macular Research Group, Room 116, Level 1, Save Sight Institute, Campus of Sydney Eye Hospital, 8 Macquarie St, Sydney, NSW 2000 Australia

**Keywords:** Patient satisfaction, Experiences, Clinical trials, Retinal, Macular

## Abstract

**Background:**

Patient-centered care recognizes the obligation to understand and meet patient’s expectations. An individual’s satisfaction has been found to affect health-related decisions and treatment-related behaviours, which in turn affect medical compliance, follow-up, the success of treatment and the appropriate use of services. We studied the expectations, experiences and satisfaction of patients who participated in clinical trials for retinal diseases at the Sydney Eye Hospital.

**Methods:**

The study was undertaken at the research clinic of the major public quaternary eye hospital in New South Wales, Australia. A 37-question survey was conducted on patients enrolled in or who had finished a clinical trial for macular disease in the 12 months preceding this study in November 2012. Patient satisfaction was assessed using close-ended, multiple choice questions. First, the decision making process for entering into the clinical trial was evaluated. Then the level of patient understanding and experience during the study was assessed. Finally, there was a series of questions to gauge the participants’ perception of trial outcomes and overall impression gained from the experience.

**Results:**

Eighty patients completed the questionnaire. Overall patient satisfaction was high with the majority of patients stating they would recommend participation in a retinal clinical trial (94 %) and participate in a subsequent trial (78 %). Most patients rated themselves as the most important factor in making the decision to join a trial. Patients felt well informed and expectations were generally felt to be met, however 14 % did not believe that they could withdraw from the study voluntarily. The most common reasons for trial participation were to contribute to medical science and to have improved treatment outcomes.

**Conclusions:**

We found that patients generally found participation in retinal clinical trials to be a positive experience. Factors contributing to dissatisfaction mainly related to inconvenience experienced by transportation and waiting times. We also found that patients felt well informed about the study, but some did not have a complete understanding of their rights, which had been communicated to them when they entered the study. There were both altruistic and self-motivated reasons behind patients’ decisions to join a retinal trial.

**Electronic supplementary material:**

The online version of this article (doi:10.1186/s12886-015-0071-6) contains supplementary material, which is available to authorized users.

## Background

Clinical trials are considered the ‘gold standard’ by which clinicians decide if treatments are safe and effective. Patient satisfaction, defined as the fulfilment of expectations and needs, incorporates patients’ perceptions and preferences when evaluating the success of medical treatments and healthcare delivery systems [1–3]. Moreover, an individual’s satisfaction has been found to affect health-related decisions and treatment related behaviors, which in turn affect medical compliance, follow-up, the success of treatment and the appropriate use of service [4–7]. Previous surveys suggest that patients positively view their experience of ophthalmology clinics [8–12]. Patient satisfaction has been linked to participation in decision-making, clinicians’ communicative behavior, treatment outcome and patients’ expectations regarding psychosocial support as well as therapeutic listening [13–20]. However, this has not been examined specifically in the context of retinal clinical trials, which may be more labour intensive than interventions for other medical conditions.

The objective of our study was to understand what motivated patients to participate in clinical trials for retinal disease, and to determine if the experience was a satisfactory one. This is important, as patient satisfaction is linked to patient compliance with therapy [21, 22], which is necessary for optimal long term outcomes, as well as increased number of hospital recommendations by patients to others and improved reputation [23, 24] We anticipated that the information gained from this study might identify ways to improve the clinical trial process and aid in patient recruitment. Information gathered about level of understanding and decision-making process is also relevant to understanding patient consent.

## Methods

### Study subjects

This was a cross-sectional study of all patients currently enrolled in, and those who had completed clinical trials for retinal disease in the preceding 12 months at the start of the study in November 2012, in the Macular Research clinics at the Sydney Eye Hospital. The Sydney Eye Hospital is a quaternary referral unit located within the Central Business District of Sydney. Participants were available from 14 retinal clinical trials. Eighty consecutive patients who were attending the research clinics were recruited for this exploratory, non-comparative study.

### Ethics

The study was approved by the Human Research Ethics Committees of Royal Prince Alfred Hospital (LNR/12/RPAH/382). Patients read the participant consent form and verbal consent was obtained by the research staff. Participation was voluntary.

### Survey administration

Research staff identified eligible patients upon their arrival at the clinic and gave them an information sheet on the study along with the questionnaire. The surveys were either self completed or were completed with the help of an accompanying friend, family member or interpreter. Large font versions of the questionnaire were available for those who were visually impaired. The survey was filled out anonymously with only generic demographic data collected. Patients returned their completed surveys to a sealed box in the clinic area.

### Data analysis

The survey format consisted of a 37 item questionnaire (Additional file 1). Questions were formulated by the authors of the present study after a literature review of previous surveys [8–20], and after discussion with medical, nursing and paramedical staff, to ensure face validity. The questions were chosen to represent a wide range of areas of concern that might affect patient satisfaction in retinal clinical trials. The development of this questionnaire was guided by the evidence base for selection of rating scales [25–27]. Rating scales had a simple question format, with categories that included frequency, severity and global ratings. These rating scale categories were presented in a clear progression and were conceptually exhaustive, using a Likert scale to scale responses allowing for one response [28, 29]. For the small number of questions that allowed for multiple responses, the percentage of each response was calculated by dividing the total number of each response by 80. Responses left blank were censored as missing data.

## Results

### Description of the clinical trials

Participants in 14 retinal clinical trials were eligible for the study. Most of the retinal clinical trials compared the outcomes of two different drugs, although some compared the effect of a drug versus a placebo. A full description of each trial is provided in Additional file 2. The survey was completed by 96 % of the eligible subjects approached. Of the three patients who declined the survey, two could not read English and one had poor vision and did not have an accompanying carer. Of the 80 participants, approximately half (53 %) were male, with the median age within the 61 and 70 age selection range. Half the patients surveyed (51 %) were born outside Australia and English was a second language for 30 % of participants. Approximately a third (36 %) of the 80 participants had been in a previous retinal trial at the Sydney Eye Hospital.

### Decision-making process and entry into the trial

The majority of patients (88 %) stated that their main source of information for entering the trial was from medical staff (doctors and nurses), and 96 % of patients thought they received adequate information about the trial. More than half of the patients (55 %) stated that they themselves were the most influential in their decisions to join the trial, while 30 % thought that doctors were the most influential. Most patients (84 %) made their decisions to participate within one day.

**Fig. 1:**
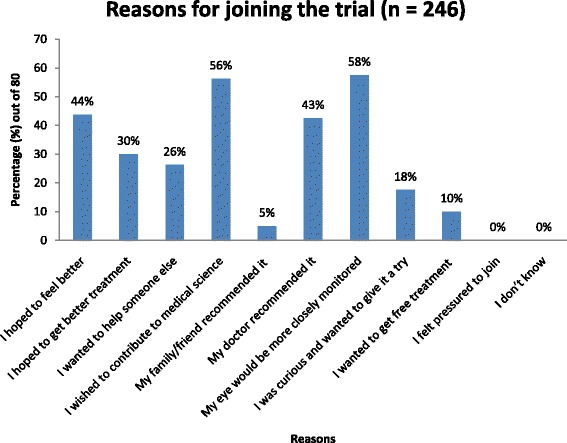
Reasons for joining a retinal clinical trial at the Sydney Eye Hospital

Reasons for participating in the trial varied, but the two most popular responses were that participants ‘wanted to contribute to medical science’ and they wanted their ‘eyes to be more closely monitored’ (Fig. 1). None of the participants felt they had been pressured to join or were unable to ascribe a reason for joining. A total of 246 responses were obtained for this question.

### Perceived benefits and problems with trial participation

Patients were also allowed multiple responses when describing perceived benefits of trial participation. The majority of patients (76 %) stated the goal of participation within their particular clinical trial was to improve medical care for future patients, half (50 %) described participation was to benefit themselves and a third (35 %) also felt that the goal was to give doctors experience with a new drug. Ninety percent of patients agreed that the trial provided important information to medical science. The vast majority of patients (95 %) agreed that staff kept them up to date on the study progress.

When asked about problems with trial participation, a sizeable minority of patients (24 %) thought too much time was spent at the clinic. Other problems highlighted included transport difficulties (13 %), parking problems (8 %), unclear directions to the appointment location (3 %), difficulty getting off work for appointments (3 %) and changes of clinical staff (1 %). Fourteen percent of patients thought they could not withdraw from the trial, which was incorrect. The majority of patients did not think there were too many follow up visits (74 %) or forms to complete (78 %).

### Trial outcomes

Subjective outcomes of participation in the clinical trials were generally positive, as outlined in Table 1. Sixty three percent of participants stated that they felt much or somewhat better as a result of participating in the study while only 4 % felt somewhat worse.

**Table 1:** Trial outcomes from the patients’ perspectives

Question	Response	n (%)
How did you benefit from participating in the trial? (N = 187 responses)	More frequent contact with my doctor	33 (41 %)
	Free medical care and services	35 (44 %)
	Remediation^a^	12 (15 %)
	More knowledge about my eye condition	68 (85 %)
	Improved health	23 (29 %)
	Interaction with others with my condition	11 (14 %)
	Other	5 (6 %)
How have you felt since having the treatment?	Much better	32 (40 %)
	Somewhat better	18 (23 %)
	About the same	15 (19 %)
	Somewhat worse	3 (4 %)
	Much worse	0 (0 %)
Have you experienced any side effects from the treatment?	Yes^b^	11 (14 %)
	No	50 (63 %)
	Unsure	5 (6 %)

^a^Remediation refers to correction of visual health as defined by the specific retinal trial

^b^Side effects listed by patients included pain, increased appetite, hair hanging in eye, headaches, cataract, blurred vision, smell, burning and gritty sensation in eye, small tingles in eye, and glaucoma

### Relationship with medical staff

Overall, patients had a positive impression of medical staff: 93 % thought staff always treated them with courtesy and respect and 96 % thought staff were always helpful. From the patient’s perspective, clinical staff always (86 %) or usually (9 %) worked well together as a team. Seventy six percent of patients always felt valued and appreciated as a patient, while a further 15 % thought that this was the case usually. Eighty six percent of patients *never* had any doubts about the ability of their treating doctors, although 6 % *sometimes* had doubts and 2 % *usually* had doubts. However, no patient *always* had doubts about their doctors’ abilities. Since enrolment in a clinical trial, the patient’s relationship with their doctor improved in over half (58 %) of cases, in 38 % the relationship remained unchanged and no patient described trial participation worsening their relationship with the treating medical team.

### Overall patient impression of the clinical trial

Table 2 outlines patients’ overall impression of their clinical trials, which was mostly positive. The overwhelming majority of patients felt that taking part in the trial was important for their condition and would recommend participation to another person.

**Table 2:** Overall impression of the retinal clinical trial

Question	Response	n (%)
How important do you feel taking part in this trial is to your condition?	Very important	68 (85 %)
	Fairly important	5 (6.25 %)
	Slightly important	3 (3.75 %)
	Not at all important	2 (2.5 %)
	Missing data	2 (2.5 %)
Would you recommend participation in this trial?	Yes	75 (93.75 %)
	No	2 (2.5 %)
	Unsure	2 (2.5 %)
	Missing data	1 (1.25 %)
Volunteer for another trial?	Yes	62 (77.5 %)
	No	3 (3.75 %)
	Unsure	14 (17.5 %)
My expectations of joining the trial were	Met	50 (62.5 %)
	Somewhat met	18 (22.5 %)
	Somewhat unmet	2 (2.5 %)
	Unmet	2 (2.5 %)
	Unsure	3 (3.75 %)
	Missing data	5 (6.25 %)
How would you rate the overall quality of care and services provided in the clinical trial?	Excellent	59 (73.75 %)
	Good	16 (20 %)
	Fair	1 (1.25 %)
	Poor	0 (0 %)
	Missing data	4 (5 %)
Would you return to the Sydney Eye Hospital should the need arise?	Yes	73 (91.25 %)
	No	0 (0 %)
	Unsure	4 (5 %)
	Missing data	3 (3.75 %)

## Discussion

Increasingly, participant experience studies are undertaken as part of clinical trials to improve recruitment, as well as the delivery and conduct of future trials [30]. Most of these studies, not in the field of ophthalmology, have focused on patients’ understandings and experiences and how these might influence recruitment, retention and adherence to the investigated intervention [31–34]. Ophthalmology patient satisfaction studies have mainly been in the areas of cataract and refractive surgeries [8–11], as well as in for oculoplastics and glaucoma surgeries [12, 35]. Our study provides insight into patient experiences and satisfaction in clinical trials in the context of translational retinal research.

In this study we surveyed 80 consecutive patients participating in various clinical trials for retinal diseases. We found that participants generally found their experience in retinal clinical trials to be positive and satisfying. These findings are reassuringly consistent with other studies of patient satisfaction within the clinical trial environment in ophthalmology as well as other fields of medicine [9, 13–15, 32, 33].

Recruiting and maintaining participants in clinical trials is vital and often challenging. In order to achieve good trial recruitment and retention, an understanding of what makes the experience satisfactory for the patient is likely to be helpful. To address this need, our study investigated the reasons for trial participation, expectations and measures of satisfaction. In a study including inpatients and outpatients after cataract surgery from three facilities, Nijkamp et al. found that satisfaction with regard to the quality of care, judgments about the counseling, and meeting patients’ preoperative expectation concerning the medical outcome were predictors of overall patient satisfaction [14]. Predictors were consistent among the investigated settings and overall satisfaction scores also did not significantly differ. Jackson et al. also reported that understanding and meeting initial expectations is an important component of achieving a satisfactory patient experience in a general medicine walk-in clinic [36].

We examined the decision making process to enter clinical trials of retinal disease in terms of patient understanding and reasoning. We found that the primary source of trial information came from medical staff, with the majority of patients (96 %) feeling that adequate information had been given to them. This emphasises that ophthalmologists and other clinical staff have an important role to increase patient participation in trials, since they are by far the primary source of information about the study. However, most patients rated themselves as the most important factor in making the decision, with the decision usually made within 24 h; this emphasizes that recruitment in clinical trials should be patient-focussed with the amount and level of information geared appropriately towards patients.

It is noted that 14 % of patients did not feel they could withdraw from the study. This is an inaccurate perception which demonstrates the potential for misunderstanding in the consent process, possibly resulting from language barriers and the use of lengthy consent documentation. Participants might have been more focused on the actual treatment and side effects or visit scheduling information and the decision making of whether to enter a clinical trial, and less on other issues such as withdrawing. The conduct of verbal consent, the conduct of the survey on site, and the signing of the consent for retinal studies all in one day may all be unintentional subtle sources of undue influence. Nevertheless, this highlights the importance of clearly explaining to patients their rights when entering the study as well as their obligations, and not ‘flooding’ patients with too much information simultaneously.

We found that there were both strong altruistic and self-motivated reasons behind patients’ decision to participate in trials. The most popular responses as to why they participated in a clinical trial were ‘to assist medical science’ and ‘to have (their) condition more closely monitored’. These findings were corroborated by measures for patient satisfaction, with the most common benefit described by patients as increased knowledge of their particular medical condition. Other popular responses were free medical care and services and increased contact with the treating team.

Consistent with other studies, there appears to be a desire amongst a significant proportion of patients to feel actively involved in their care, expressed by a desire for greater knowledge and greater contact with staff [37]. This increased involvement can lead to improved patient outcomes as well as satisfaction. It is acknowledged that those who had volunteered for trials were more likely to have this desire for participation. In terms of advocating trial involvement, it can be seen from our study that those who joined largely described a desire for a greater sense of knowledge, involvement and frequency of care, and that expectations were largely satisfied through the trial process.

Another aspect of assessing participant satisfaction was the patient response to services and caregivers. We found that the vast majority of patients within the retinal research clinics studies were very satisfied with services and staff, with 74 % rating them as ‘excellent’. Previous studies have consistently demonstrated the importance of communication between patients and their caregivers and the value of providing relevant information such as regarding operative processes or diagnostic tests [16, 38–43]. Our study corroborated these findings, with patient interaction with staff likely playing an important role in achieving overall high satisfaction. The impact of study participation on relationship with staff was also demonstrated by the fact that 58 % of patients thought their relationship with their doctors improved through participating in the clinical trial. Further qualitative research may be warranted to elucidate the specific important aspects of this relationship with patients. A qualitative study using focus groups by Dawn et al. identified 6 areas of expectations for eye care that were important to patients: honesty, information about diagnosis and prognosis, explanation in clear language, ophthalmologists’ experience and reputation, empathy and listening and addressing concerns [19].

We also sought to identify areas of dissatisfaction in order to understand if these could be addressed to improve the patient experience and whether these elements of dissatisfaction were related to the trial experience. The main problem identified was prolonged wait time in the clinic, transport and parking problems. Communication and information provision to health consumers, especially in relation to waiting times, have been shown to have positive effects on satisfaction levels, resulting in significant falls in complaint levels [44]. A discussion and briefing regarding wait times is indeed performed with patients in the clinics studied. While wait times remain a source of discontent, it is possible that the communication that takes place around these times mitigates their impact on overall patient satisfaction, reflected in the fact that 93 % of patients remained willing to return to the public outpatient clinics at the conclusion of the study. Further, patients did not feel their time was being generally wasted, as three-quarters of them did not think there were too many follow up visits or that there were too many forms to complete. The latter may be because data collection was predominantly performed by study staff rather than patients. There were very few studies that asked patients to complete forms.

Access to the clinic was a major cause of patient dissatisfaction. Since the hospital is a quaternary referral service, as is likely to be the case for other centres where clinical trials of retinal disease are performed, patients usually were not travelling from the immediate locality. Sponsored clinical trials would have the means to provide adequate reimbursement for travel and parking to the investigational site, but this may not be feasible for investigator initiated trials.

The major limitations of this study are those inherent in cross-sectional, ‘self-reporting’ questionnaire surveys. Patients who are more satisfied with care are less likely to return questionnaires, thus potentially under-estimating satisfaction levels [45]. Despite this, the response rate of our survey was high at 96 %, with those few declining mainly citing language difficulties. Nevertheless, we could not assess the differences in baseline or clinical characteristics between the responders and non-responders. Furthermore, there was the potential undue influence of patients being handed the survey by research staff and the expectation to complete it on site. A lower response rate might have been attained if the survey was not allowed to be completed on site, however this approach would cause greater inconvenience to patients and would yield a much lower response rate. Another limitation of this study was that the distribution of patients across the 14 retinal trials was not known, as we did not collect this information from the questionnaire. We confirmed that they were from at least one of the listed studies only. Our population was a consecutive sample and included patients from a number of different trials. Our clinics are not differentiated based on which clinical trials the patients are part of.

Tendency for respondents to bias towards positive responses and use acquiescent replies was minimized by adopting positively or negatively worded, specific questions [25, 46, 47]. However, one question was constructed with a positive bias: “How did you benefit from participating in the study?” This question did not offer a neutral or negative response option, and thus it was unsurprising that a high majority of respondents described this study as beneficial. This could have been avoided if a more vigorous question selection process had been undertaken, that would include multiple pilot tests, focus groups and interviews to enhance content validity [48, 49]. Other questions appropriately included the full range of response options. In addition, while the specific and limited range of responses allowable assisted in giving a good overview of quantifiable data, further qualitative research would likely be of benefit to explore further patient views and level of understanding. It is conceded that if we had studied patients who were approached for participation in clinical trials rather than those who had already consented to join, we may well have found different responses to some questions.

## Conclusions

Patient satisfaction is of fundamental importance as a measure of the quality of care because it gives information on the providers’ success at meeting health consumers’ values and expectations. The results of this study indicate that most patients were satisfied with their experiences and outcomes from participating in a retinal clinical trial at the Sydney Eye Hospital, despite dissatisfaction with some aspects of the clinical trial process and a small percentage of patients experiencing some side effects. Gaining a better understanding of patients’ expectations in ophthalmic clinical trials may help better guide efforts to educate patients, to reduce unreasonable expectations and, ultimately, to improve their experience.

## Additional files

Additional file 1:
**The study questionnaire.** (DOCX 138 kb) Additional file 2:
**A brief description of each clinical trial.** (DOCX 82 kb)
