# Supplementary material for: Patient experiences in retinal trials: a cross-sectional study
Source: BMC Ophthalmol. 2015 Jul 23;15:80. doi: 10.1186/s12886-015-0071-6 (PMC4511246; doi:10.1186/s12886-015-0071-6)
Supplement: Additional file 1: — The study questionnaire. (DOCX 138 kb) [file 12886_2015_71_MOESM1_ESM.docx]

**APPENDIX 1: The study questionnaire**

| **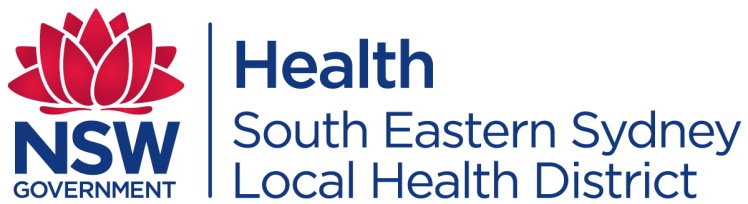** |
| --- |

***Patient Experiences in Retinal Trials – PERT Questionnaire***

***Thank you for your participation in a retinal clinical trial at the Sydney Eye Hospital. We would like to learn more about your experience with us. It will take approximately 10 minutes to complete this form. Results will remain anonymous. Please answer all the questions and return it to the box at the front desk.***

## About you

| 1. Gender   - Male - Female   2. Age   - 18-30 - 31-40 - 41-50 - 51-60 - 61-70 - 71-80 - Over 81 | 3. Which country were you born in?  _______________________________  4. Which country was your mother born in?  _______________________________  5. Which country was your father born in?  _______________________________  6. What is your first language?   - English - Other (______________________) |
| --- | --- |

## About the clinical trial

| 7. Have you previously participated in a clinical trial?   - Yes - No - Unsure   8. Would you recommend participation in this trial?   - Yes - No - Unsure   9. Were you told what to expect from participating in the trial?   - Yes - No - Can’t remember   10. What was your main source of information about the trial?   - Medical staff - Posters/booklets - Relatives/friends/former trial participants - Mass media - Other (please specify ______________)   11. Did you receive enough information before the trial?   - Yes - No - Unsure   12. What were your reasons for joining the trial? (can tick more than 1)   - I hoped to feel better - I hoped to get better treatment - I wanted to help someone else - I wished to contribute to medical science - My family/friend recommended it - My doctor recommended it - My eye would be more closely monitored - I was curious and wanted to give it a try - I wanted to get free treatment - I felt pressured to join - I don’t know - Other (please specify ______________________)   13. Who was the most influential person in your decision?   - Myself - Family/friend/relative - Doctor - Nurse/other medical staff   14. How long did you take to make the decision to join the trial?   - Within 1 day - More than 1 day   15. How did you benefit from participating in the trial?  (can tick more than 1)   - More frequent contact with my doctor - Free medical care and services - Remediation - More knowledge about my eye condition - Improved health - Interaction with others with my condition - Other (__________________)   16. The goal of the trial is to   - Benefit the patient - Improve medical care for future patients - Give the doctor experience with new drug - Other (please specify____________________)   17. The trial drug is being compared to   - Another drug - Placebo   18. How have you felt since having the treatment?   - Much better - Somewhat better - About the same - Somewhat worse - Much worse   19. Have you experienced any side effects from the treatment?   - Yes (please specify______________________) - No - Unsure   20. Would you volunteer for another research trial?   - Yes - No - Unsure   21. Did you have any problems with the clinic? (can tick more than 1)   - Transport problems - Parking problems - Inaccessible to handicapped patients - Directions to facility unclear/confusing - Directions to appointment location unclear - Difficulty getting off work for appointments - Clinic environment unclean - Unpleasant clinic atmosphere - Clinical staff personnel changes - Not enough information about trial - Inconvenient scheduling of visits - Hurried clinic visits - Too much time spent at clinic - Other (please specify____________________)   22. How important do you feel taking part in this trial is to your condition?   - Very important - Fairly important - Slightly important - Not at all important   23. The staff kept me up to date on the trial progress   - Strongly agree - Agree - Disagree - Strongly disagree   24. The trial provided important information to medical science   - Strongly agree - Agree - Disagree - Strongly disagree   25. There were too many follow-up visits   - Strongly agree - Agree - Disagree - Strongly disagree   26. There were too many forms to complete   - Strongly agree - Agree - Disagree - Strongly disagree   27. Would you be free to withdraw from the trial at any time?   - Yes - No - Unsure   28. Did you want to withdraw from the trial?   - Yes - No - Unsure   29. My expectations of joining the trial were   - Met - Somewhat met - Somewhat unmet - Unmet - Unsure |
| --- |

## About the clinical trial staff (doctors, nurses, receptionist, etc)

| 30. How often did the staff treat you with courtesy and respect?   - Never - Sometimes - Usually - Always   31. How often were staff as helpful as you thought they should be?   - Never - Sometimes - Usually - Always   32. Overall the clinical staff seemed to work well together as a team.   - Never - Sometimes - Usually - Always   33. How often did you feel valued and appreciated as a patient?   - Never - Sometimes - Usually - Always   34. Did you have doubts about the ability of the doctors who treated you?   - Never - Sometimes - Usually - Always   35. Since you enrolled in the trial, your relationship with your doctor has   - Improved - Not changed - Worsened - Not sure |
| --- |

## Overall

| 36. How would you rate the overall quality of care and services provided in the clinical trial?   - Poor - Fair - Good - Excellent | 37. Would you return to the Sydney Eye Hospital should the need arise?   - Yes - No - Unsure |
| --- | --- |
