# Supplementary material for: Patient experiences in retinal trials: a cross-sectional study
Source: BMC Ophthalmol. 2015 Jul 23;15:80. doi: 10.1186/s12886-015-0071-6 (PMC4511246; doi:10.1186/s12886-015-0071-6)
Supplement: Additional file 2: — A brief description of each clinical trial. (DOCX 82 kb) [file 12886_2015_71_MOESM2_ESM.docx]

**APPENDIX 2: A brief description of each clinical trial**

BEVORDEX - A 2 year, phase II, prospective, multicentre, randomised, single-masked clinical trial of sustained release intravitreal dexamethasone (Ozurdex™) versus intravitreal injections of bevacizumab (Avastin®) for diabetic foveal oedema that persists or recurs despite previous laser treatment, or for which the investigator believes laser treatment is unlikely to be helpful, with either a 3 or 9 month safety follow-up from the last treatment.

FOVEA - A 6-month, phase II, double-masked, multicenter, randomized, placebo-controlled, parallel group study to assess the safety and efficacy of topical administration of two concentrations of FOV2304 (1% and 2%) twice daily for the treatment of center-involving clinically significant macular edema associated with diabetic retinopathy.

BDPRVO - A 12-Month, multicenter, 2-stage (open label, dose-escalation, followed by masked, randomized) single dose study of the safety and efficacy of intravitreal beclomethasone dipropionate (AGN-208397) in patients with macular oedema (ME) associated with retinal vein occlusion (RVO).

VIVIDDME - A randomized, double masked, active controlled, phase III study of the efficacy and safety of repeated doses of intravitreal vascular endothelial growth factor(VEGF) Trap-Eye in subjects with diabetic macular oedema.

GATE - A prospective, multi-center, double-masked randomized study with a minimum treatment of 30 months. The objective was to evaluate the safety and efficacy of a 5-HT_1A_ agonist (AL-8309 Ophthalmic Solution) versus placebo administered as a topical ocular drop for the treatment of geographic atrophy secondary to aged-related macular degeneration (AMD).

852 - A Phase IIb, randomized, multicenter, double-masked, active-controlled dose-ranging study in adults with choroidal neovascularization (CNV) secondary to age-related macular degeneration (AMD) who have responded to anti-VEGF intravitreal injections (IVT) in the study eye. The primary objective was to evaluate if pazopanib eye drops can maintain or possibly improve vision, while reducing the continued need for IVT injections of ranibizumab.

POSURDEX DME - A 3-year, phase III, multi-center, masked, randomized, sham-controlled trial to assess the safety and efficacy of 700µg and 350µg dexamethasone posterior segment drug delivery system (DEX PS DDS) applicator system in the treatment of patients with diabetic macular oedema.

BRIGHTER - A 24-month, phase IIIb, open-label, randomized, active-controlled,3-arm, multicenter study assessing the efficacy and safety of an individualized, stabilization-criteria-driven pro re nata (PRN) dosing regimen with 0.5-mgranibizumabintravitreal injections applied as monotherapy or with adjunctive laser photocoagulation in comparison to laser photocoagulation in patients with visual impairment due to macular edema secondary to branch retinal vein occlusion (BRVO).

Crystal - A 24-month, phase IIIb, open-label, single arm, multicenter study assessing the efficacy and safety of an individualized, stabilization criteria-driven pro re rata (PRN) dosing regimen with 0.5-mg ranibizumab intravitreal injections applied as monotherapy in patients with visual impairment due to macular edema secondary to central retinal vein occlusion (CRVO).

Darapladib - A phase 2 double-masked, randomized, placebo controlled, parallel-group study to investigate the safety, tolerability, pharmacodynamics and efficacy of darapladib administered for 3 months to adult subjects with clinically significant diabetic macular oedema with centre involvement.

REACH Stage 2 - A phase II, 2-stage study of approximately 200 patients with oxidative age-related macular degeneration (AMD) at approximately 40 sites. Stage 1 is an open-label dose escalation assessment of the safety of AGN-150998administered as an intravitreal injection to patients with oxidative AMD. Stage 2 is a randomized double-masked comparison of the safety and effects of treatment on retinal edema and BCVA of AGN-150998 and ranibizumab in treatment-naïve patients with oxidative AMD.

MacTel - This study collected in-clinic data on subjects for 5 years based on the date the subject was enrolled in the Natural History Observation (NHO) Study. This data collection focused on enhancing the natural history and progression of macular telangiectasis type 2 and the disease pathology through collection of post-mortem eye tissue and compiling information on a possible genetic association.

Brimo33D - A multi-center, patient-masked, safety extension study to evaluate the biodegradation of the brimonidine tartrate posterior segment drug delivery system (Brimo PS DDS) Implant up to 36 months.

RestoreEXT - An open-label, multi-center, 24-month extension study to evaluate the safety of ranibizumab as symptomatic treatment for visual impairment due to diabetic macular edema inpatients who have completed the RESTORE trial (initial 12-month treatment).
